# Pamela Emmerling: Ärztliche Kommunikation; Als Erstes heile mit dem Wort ....

**DOI:** 10.3205/zma000968

**Published:** 2015-08-17

**Authors:** Anne Simmenroth-Nayda

**Affiliations:** 1 Georg-August Universität Göttingen, Institut für Allgemeinmedizin, Lehrkoordination, Göttingen, Deutschland

## Recension

Pamela Emmerling, an educator and psychotherapist, has been active for many years as coach and university instructor in connection with the field of medicine (medical associations, medical training courses, hospital and practice consulting, etc.). Her new book, *Ärztliche Kommunikation*, is not only suitable for those working in hospitals, but also those in private practice. Although patient simulation is indeed mentioned (albeit as though this method were applied exclusively in Berlin), this book has not been explicitly written for teachers in medical education. The author’s primary focus is on providing a complete survey of all areas which are necessary for “good communication” in a medical setting. As such, this book covers many aspects that could also also adressed outsiede of medical education..

The book is divided into sections dedicated to the basic principles of communication and its models, first in terms of the patient and his or her horizon of understanding, then in terms of physicians with their various roles and possibilities, and moving on to cover the communication challenges faced by teams and finally “advanced communication”. A very detailed table of contents, effective sequencing of the sections, many case examples, highlighted key sentences, and easy-to-read diagrams and tables supplemented with a few cartoons, make this book enjoyable reading.

A large number of communication models and strategies are presented – the variety can be alarming, but also encourages the reader to engage more closely with selected models or to cover these in more detail in the classroom – modified to include example cases or role playing.

One example is the Riemann Thomann model that depicts not just the patient, but also the doctor, as a person caught between the poles of duration versus change and distance versus closeness. Many typical communicative and behavioral patterns can be interpreted using this model. Another example is taken from Transaction Analysis with its modes of “child”, “parent” and “adult” and can be applied to medicine for role playing activities or patient simulation. Even special exercises on reformulating accusations into wishes (particularly in teams) are manifold and can be applied meaningfully by all readers.

Even identifying one’s own of the five drivers (be perfect, hurry up, try hard, please me, be strong) using a psychometric test is a helpful tool for encouraging self-reflection. However, the obsolete and scientifically unfounded models, such as that of Schulz von Thun, could certainly be foregone.

No citations of the scientific literature are made; however, the references include extensive recommendations for further reading. The short pro and con statements by Dr. No and Dr. Will at the end of each chapter seem redundant and appeared to the reader as a kind of moral finger pointing that does not correspond with the well-founded content of the book itself – this could certainly be dropped from a later edition.

Even though the author’s stance on testing communicative skills using simulation is not so positive (as a part of the energy goes into the awareness of the simulation), she is able to explicitly include the current circumstances behind doctor-patient consultations (lack of time, cost pressure, pressure to succeed) and to make realistic, not impossible demands: begin by addressing small things about one’s self, make use of a “trick” or two, or simply listen more than talk! Overall, this book is extensive, but worthwhile reading for all who are clinically active or teach social and communicative competencies in the medical context.

## Competing interests

The author declares that she has no competing interests.

